# Sterile Neutrophilic Dermatosis (Sweet's Syndrome) Associated With Systemic Inflammatory Response Syndrome in a Maltese Dog: A Case Report

**DOI:** 10.3389/fvets.2022.837942

**Published:** 2022-03-21

**Authors:** ARom Cho, Hyeona Bae, Sunwoo Shin, Youngju Kim, Yeseul Jeon, Jae-Eun Hyun, Kyu-Woan Cho, Dong-In Jung, Dae Young Kim, DoHyeon Yu

**Affiliations:** ^1^College of Veterinary Medicine, Gyeongsang National University, Jinju, South Korea; ^2^College of Veterinary Medicine, University of Missouri, Columbia, MO, United States

**Keywords:** sterile neutrophilic dermatosis, Sweet's syndrome, systemic inflammation, C-reactive protein, plaque, erosions

## Abstract

We report a rare case of sterile neutrophilic dermatosis (Sweet's syndrome) accompanied by systemic inflammatory response syndrome. A 5-year-old, neutered male Maltese dog presented with extensive crusts on the whole-body surface and multifocal erosions and plaques on the four limbs. The lesions had been present for two months and did not respond to antibiotics before the presentation. In addition, the dog was lethargic, anorexic, and febrile, with joint swelling. A clinicopathologic analysis revealed neutrophilic leukocytosis with left shift and increased C-reactive protein level. Furthermore, a histopathological examination showed moderate to severe inflammatory infiltrates consisting predominantly of neutrophils from the superficial to the deep dermis. There was no evidence of bacterial or fungal infections, and autoimmune diseases, such as pemphigus, systemic lupus erythematosus, and erythema multiforme, were excluded. Sweet's syndrome, a rare skin disorder, associated with systemic inflammation was diagnosed, and the cutaneous lesions and systemic inflammation disappeared after prolonged steroid administration.

## Introduction

Sweet's syndrome (SS) is a rare condition characterized by a constellation of clinical symptoms, physical features, and pathologic findings, including fever, neutrophilia, and tender erythematous skin lesions (papules, nodules, and plaques). It also presents diffuse infiltration of mature neutrophils typically located in the upper dermis (1). Despite hundreds of reports of SS in humans in the literature, its various symptoms and etiology are not entirely understood. SS is rare in veterinary practice; in dogs, this disorder is characterized by skin lesions similar to other papules, pustules, or ulcerative to erosive lesions that suddenly occur, with other unpredictable extracutaneous involvement (2).

The systemic inflammatory response syndrome (SIRS) is a clinical condition caused by widespread inflammation, secondary to infections or sterile inflammatory diseases (3). Multiple organ dysfunction syndrome, a severe complication of SIRS, can be incited without proper intervention. Several reports postulate that SIRS and multiple organ dysfunction syndrome develop as a rare and fatal complication of SS in humans (4–6). Herein, we report the case of a dog showing skin lesions that met the criteria for SS without evidence of infection, accompanied by SIRS; the lesions disappeared completely after steroid therapy.

## Case Description

**Figure 1:**
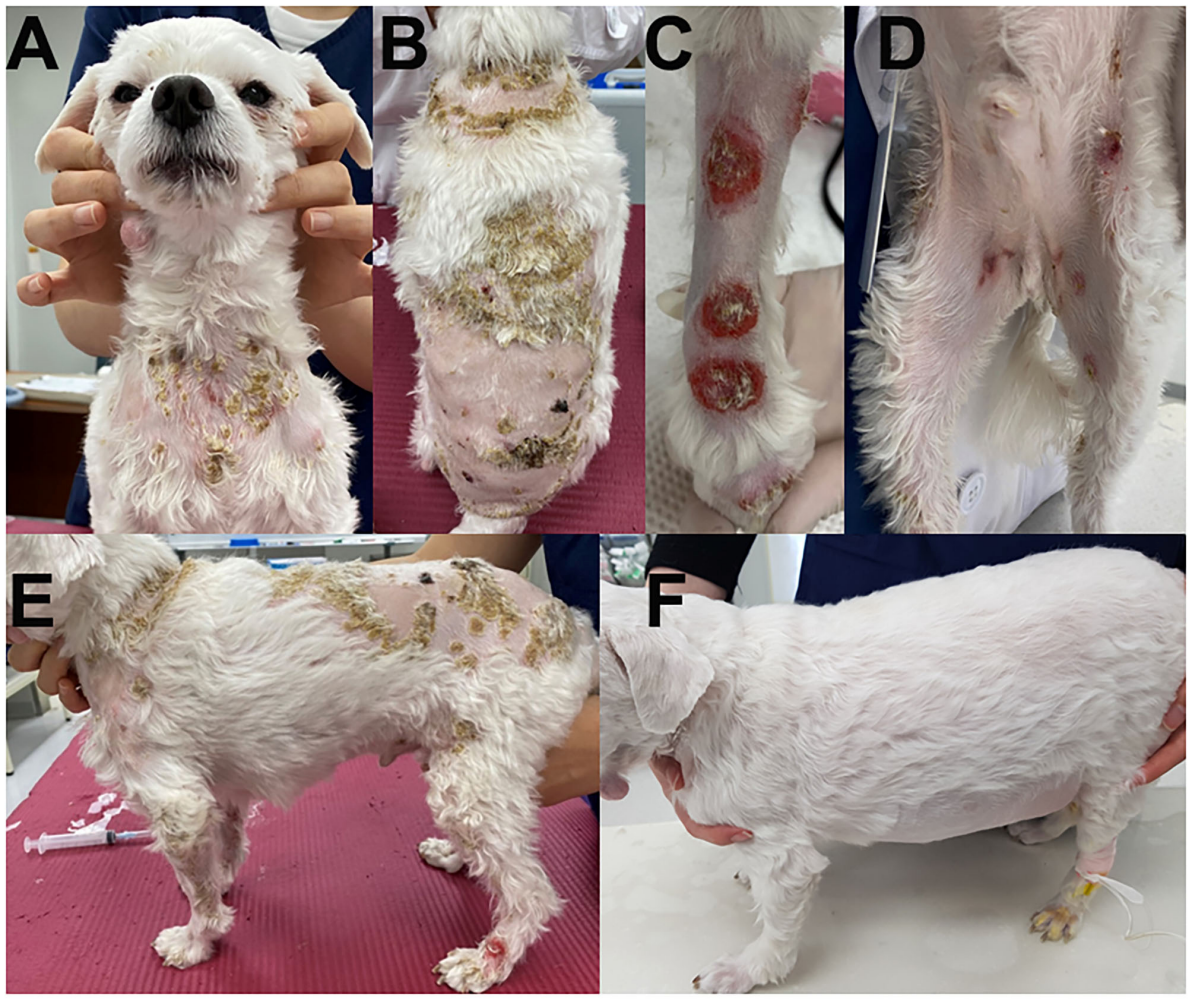
Clinical images of the skin lesions. Crusts were present on the neck **(A)** and dorsal trunk **(B)**; clearly demarcated plaques on the forelimbs **(C)**, and multiple ulcerations on the inguinal region **(D)**. Generalized skin lesions are shown on admission **(E)** and after steroid treatment **(F)**.

**Figure 2:**
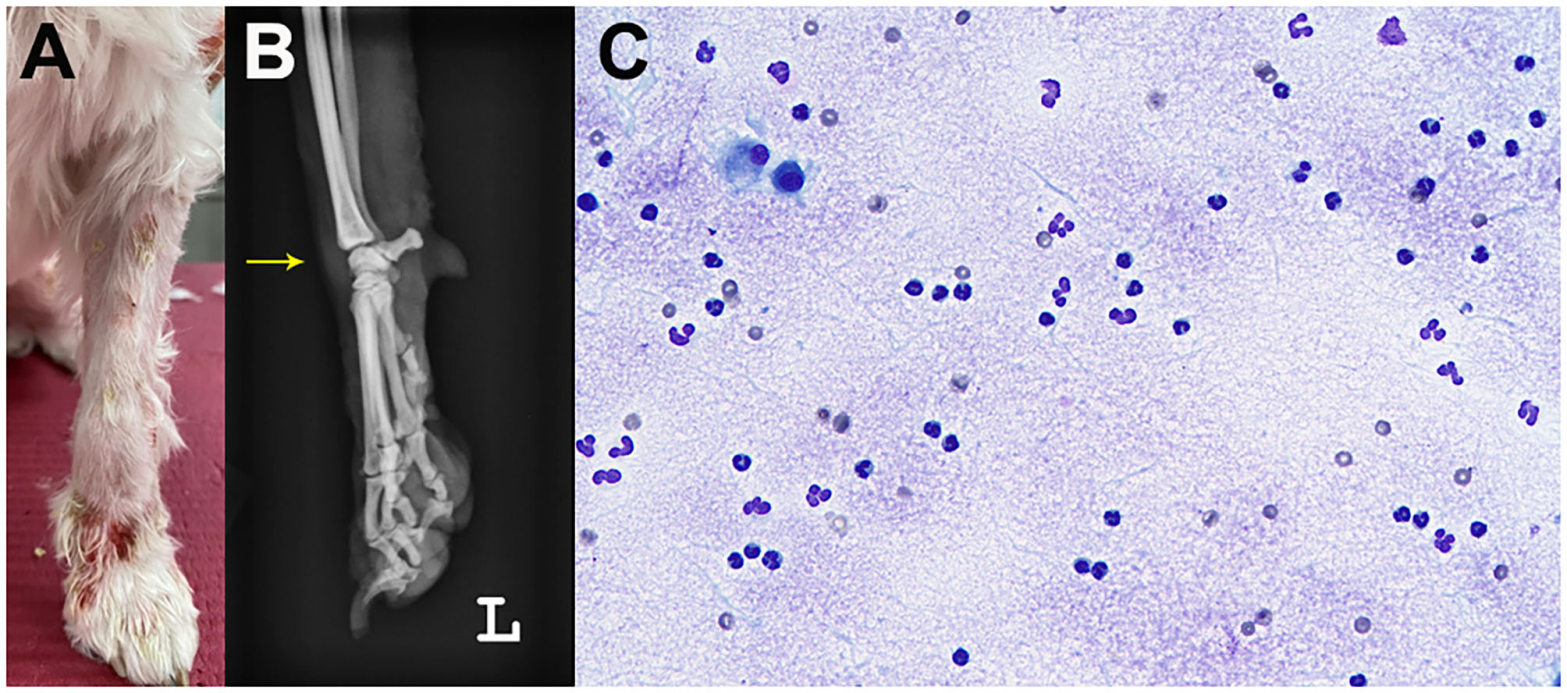
Joint swelling (arrow) of the left carpal joint. Gross image **(A)** and radiography **(B)**. Cytology of the synovial fluid showed predominantly non-degenerate neutrophils without infectious agents on synovial fluid cytology **(C)**.

A 5-year-old, neutered male Maltese dog presented non-pruritic multifocal skin lesions distributed to almost the whole-body surface. The lesions involved the face, neck, four limbs, dorsal trunk, and the perineal region, with multiple ulcerations on the back and inguinal region; clearly demarcated plaques were also observed on the forelimbs (Figure 1). The ear pinnae, oral mucosa, nose, tail tip, and digital pads were spared. The lesions were initially noticed as multiple pustules on the trunk 2 months ago; they subsequently developed into crusts and spread to the whole body without alopecia. The dog had never experienced any dermatologic issues, such as atopic dermatitis, cutaneous adverse food reaction, or drug reaction. There was no recent vaccination and medication history prior to visiting the local veterinary clinic. The skin lesions did not improve following treatment with systemic broad-spectrum antibiotics (amoxicillin–clavulanic acid, 12.5 mg/kg BID) for 2 weeks after the onset of skin lesions. Furthermore, new papules, pustules, and even ulcerations continuously appeared. In addition, the dog was lethargic, anorexic, and febrile (39.7°C) for a week before presentation and was observed to have generalized lymphadenopathy on admission. The dog lived only indoors and was regularly prevented from ectoparasites. General dermatological examinations and comprehensive laboratory tests were performed to investigate possible infectious causes or systemic diseases. Ten days after the first visit, the dog presented with left forelimb lameness and reluctance to walk; therefore, radiographic imaging and synovial fluid analysis were performed after noticing left carpal joint swelling on the third visit (Figure 2).

## Diagnostic Assessment

Cytological examination of a fine-needle aspiration samples and impression smears collected from multiple lesions showed predominantly non-degenerate neutrophils (80–90%) without infectious organisms. Although keratinized epithelial cells were exfoliated in sheets, they showed no cytological atypia. No acantholytic keratinocytes or neoplastic cells were observed. Further, no ectoparasites were observed on the skin scraping test, and fungal skin diseases were excluded by fungal culture. The lesions and fever were not responsive to cefovecin (8 mg/kg SC), which has an antimicrobial effect for approximately 2 weeks. A noninfectious etiology of the skin lesions was suspected, and multiple skin biopsies were performed.

**Figure 3:**
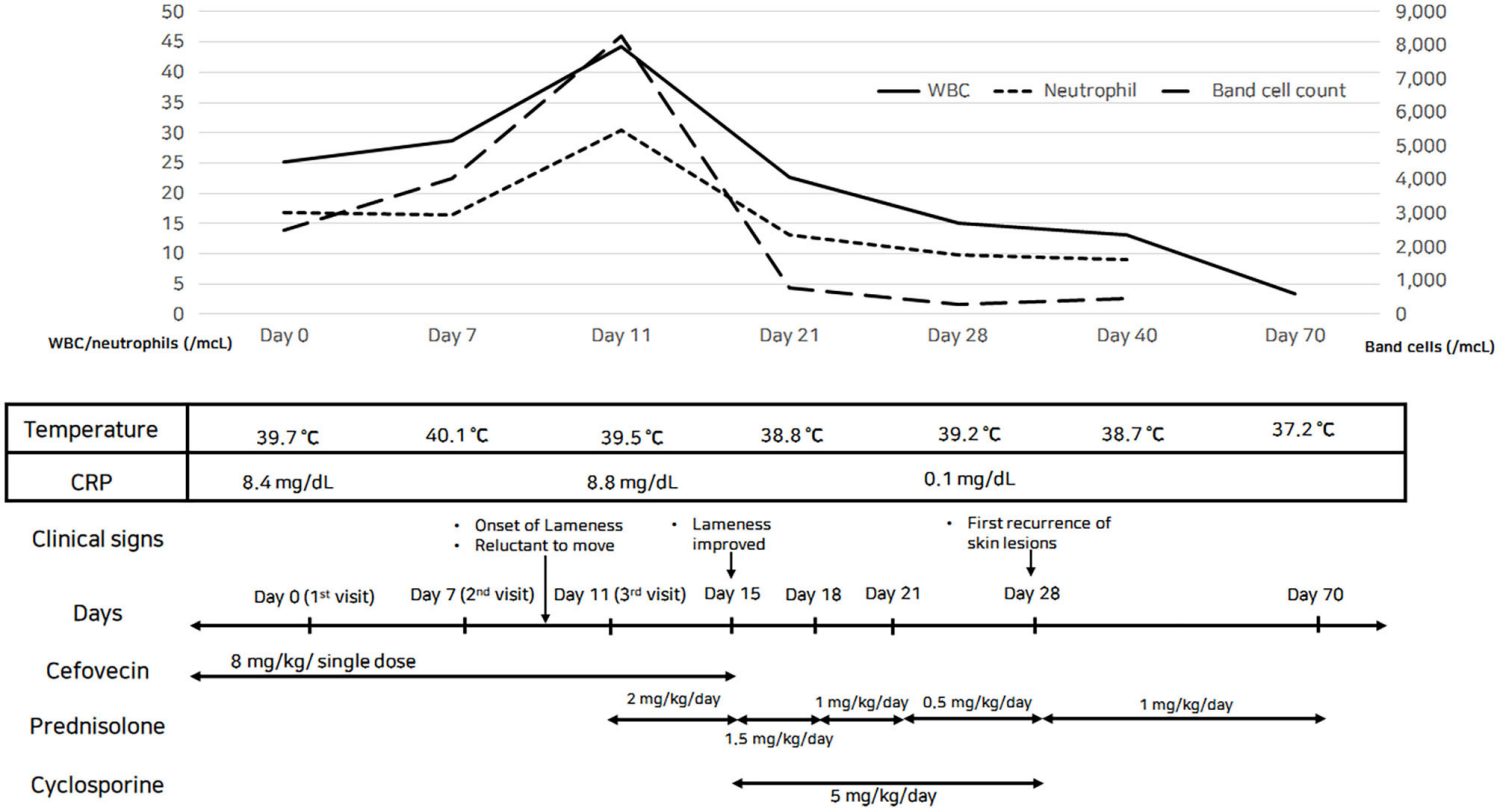
Timings of laboratory data and therapeutic trials of the dog with sterile neutrophilic dermatosis. Increased WBC count and band cell count with persistent fever indicated worsening systemic inflammation; these signs were absent after starting the steroid therapy.

Laboratory tests for systemic clinical signs showed marked leukocytosis (25.08 × 10^9^/L; reference range: 5.05–16.76 × 10^9^/L) with left shift (2,499 cells/μL, Figure 3) and increased C-reactive protein level (CRP, 8.4 mg/dL; reference range: 0.1–10 mg/dL). Fever (39.7°C), tachycardia (heart rate [HR] of 210 beats per minute), and leukocytosis with left shift (> 5% band neutrophils) met the criteria for SIRS (two or more of the following criteria need to be met for a diagnosis of SIRS in dogs: hypothermia [core temperature < 37.8°C] or hyperthermia [core temperature > 39.7°C]; tachycardia [HR > 160 beats per minute]; tachypnea [respiratory rate > 40 breaths per minute]; and leukocytosis [White blood cell count (WBC) > 12,000/uL], leucopenia [WBC < 4,000/uL], or left shift [> 10% band neutrophils]) (7). Ehrlichiosis, anaplasmosis, and Lyme disease were excluded using the 4Dx SNAP kit (IDEXX Laboratories, Westbrook, ME, USA), and additional fine needle aspiration of multiple lymph nodes revealed reactive lymphadenopathy. A synovial fluid analysis of samples from the left carpal and elbow joints indicated immune-mediated polyarthritis, with a total cell count of 3.29 × 10^9^ cells/L (reference range: < 3 × 10^9^ cells/L), increased total protein (4.5 g/dL; reference range: 2.5–3.0 g/dL) with abundant non-degenerated neutrophils (more than 12%), and a few mononuclear cells without infectious organisms (Figure 2). An antinuclear antibody (ANA) test and rheumatoid factor were negative.

**Figure 4:**
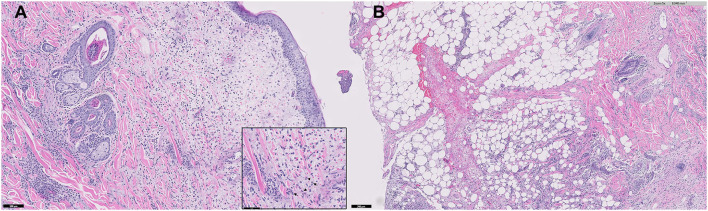
Microphotograph of a skin biopsy. Remarkable infiltration of neutrophils (arrows) from the superficial to deep dermis **(A)** and also multifocally extending to the subcutaneous tissue **(B)**.

A histopathological examination of multiple skin biopsies using 6 mm diameter punch revealed similar alterations; diffuse from the superficial to the deep dermis and multifocally extending to the panniculus, moderate to focally marked inflammatory infiltrates were present, consisting predominantly of viable neutrophils, with few lymphocytes, plasma cells, macrophages, and rare mast cells (Figure 4). The affected small-caliber vessels were filled with neutrophils that occasionally exhibited transmigration; however, most vessels within the sections were intact. There was moderate dermal edema and epidermal hyperplasia. Sebaceous glands were visible, and anagen bulbs were prominent. No dyskeratotic cells, acantholytic cells, or lichenoid band of inflammation were noted. Primary neoplasia and autoimmune skin diseases, such as erythema multiforme and pemphigus, were excluded by histopathology. Grocott's methenamine silver stain and Wright stain did not reveal fungal or bacterial elements. The skin biopsies showed moderate to severe, superficial to deep neutrophilic, interstitial dermatitis consistent with canine sterile neutrophilic dermatosis (SS). Based on the histopathologic findings, additional diagnostic workups, and clinical course, the dog was diagnosed with sterile neutrophilic dermatosis, resembling SS in humans, accompanied by SIRS and polyarthritis as extracutaneous manifestations.

After a trial with an immunosuppressive dose of oral prednisolone (2 mg/kg/day) for 5 days, the fever disappeared, the dog was no longer limping, and his general condition improved. After confirming the therapeutic effect of prednisolone, it was tapered to 3/4 of the original dose (1.5 mg/kg/day) for 3 days, and cyclosporine (5 mg/kg/day) was added for long-term management. After that, the dose of prednisolone was tapered continuously in stages to half the original dose (1 mg/kg/day) for 3 days and 1/4 of the original dose (0.5 mg/kg/day) for seven days. However, plaques and ulcerations recurred in the flank region during the cyclosporine induction period (two weeks after starting cyclosporine therapy); therefore, we interrupted this drug and returned to prednisolone monotherapy, up-titrated to 1 mg/kg/day for 6 weeks, and the clinical signs were well managed. The dog showed a dramatic improvement in SIRS, and the leukogram returned to the reference interval (Figure 3). The skin lesions on the dorsal trunk and limbs started to resolve as fever and lameness disappeared, without concurrent administration of antibiotics. The multifocal small ulcerations and plaques on the dorsal trunk healed without scarring or crusting.

Finally, the cutaneous lesions were not observed 2 months after the first presentation; therefore, prednisolone was slowly tapered (Figure 1).

## Discussion

Sterile neutrophilic dermatosis in dogs is an extremely rare disease comparable to human SS, also called acute febrile neutrophilic dermatosis (8). The diagnostic criteria for the classical SS were originally proposed by Su and Liu in 1986 and modified by von den Driesch in 1994 (1). The SS diagnosis is based on the presence of two major criteria (abrupt onset of painful erythematous plaques or nodules and histopathologic evidence of a dense neutrophilic infiltrate without leukocytoclastic vasculitis) and two of the following minor criteria: (1) fever; (2) associated underlying malignancy, inflammatory disease, or pregnancy; (3) excellent response to systemic corticosteroid therapy; and (4) hematologic abnormalities at presentation (leukocytosis, neutrophilia, or increased CRP) (1). Although diagnostic criteria for SS have not been established in veterinary medicine, human medicine criteria might be applied to diagnose sterile neutrophilic dermatosis in dogs, as these two diseases share similar characteristics; however, infections should be excluded through bacterial and fungal culture or histologic examination if infectious etiology is suspected. This case presented the two major and two minor criteria (fever and responsiveness to steroids) based on the criteria for SS in humans.

In this case, considering the clinical signs (extensive skin lesions, reactive lymphadenopathy, and immune-mediated polyarthritis) and laboratory abnormalities (leukocytosis with left shift and increased CRP level), the differential diagnosis included infectious/septic vasculitis, pustular dermatitis, neoplasia, or immune-mediated skin diseases, systemic lupus erythematosus, adverse drug reaction, pyoderma gangrenosum, erythema multiforme, and sterile pyogranuloma/granuloma syndrome. Skin infectious causes were excluded by cytology, fungal culture, and special histopathologic stains. Cutaneous neoplasia, including leukemic cutis, erythema multiforme, vasculitis, and sterile pyogranuloma/granuloma syndrome, were also eliminated by histopathological examination. Pyoderma gangrenosum shares the phenomenon of pathergy with SS; however, this disorder is clinically distinguished from SS by the presence of ulcerative lesions with an undermined, violaceous edge and cribriform scar after healing (9). Systemic lupus erythematosus was unlikely in this case because the dog did not show any other clinical signs of lupus except for polyarthritis and negative ANA titer dose not satisfied the SLE diagnostic criteria, nor the histological features. The dog was neither vaccinated in the previous year nor prescribed common drugs known to cause adverse drug reactions. The systemic inflammation progressively worsened despite antibiotic therapy, and polyarthritis appeared before initiating steroid therapy. Considering this evidence and the therapeutic response, we concluded that the cutaneous and extracutaneous manifestations corresponded to SS in humans.

In humans, this syndrome is characterized by pyrexia, neutrophilia, and tender erythematous skin lesions that show a dense neutrophilic dermal infiltrate, mostly occurring on the upper extremities, head, and neck (1). In addition, extracutaneous manifestations of SS may be present in the bones, central nervous system, ears, eyes, kidneys, intestines, liver, heart, lung, mouth, muscles, and spleen (1). Several cases of canine sterile neutrophilic dermatosis have been reported, including this case; similar to humans, extracutaneous symptoms were observed frequently in veterinary cases (2, 10–15). In addition, several cases reported lameness before or after the onset of skin lesions (2, 10, 11, 14) as well as death from pneumonia or cardiac failure within days of fever or skin lesion onset (11). Exophthalmos and periorbital inflammation have also been reported in dogs as ocular SS manifestation (13).

Fever is the most frequent symptom and is present throughout the episode of dermatosis (1). Similar to human reports, fever was noted in most veterinary cases; however, this did not always simultaneously occur with the skin lesions (2, 10, 11, 13–15). Another common extracutaneous sign is neutrophilia (2, 11, 13–15). Although neutrophilia and fever are common findings, it is unclear whether SS causes SIRS. In veterinary medicine, SIRS is diagnosed when two or more of the following criteria are met in dogs: body temperature ≤37.8°C or ≥ 39.7°C, heart rate ≥ 160 beats per minute, respiratory rate ≥ 40 breaths per minute, and WBC count ≥ 12,000 or ≤4,000 WBCs/μL, or immature (band) forms of neutrophils > 10% (7). However, in this case, fever, leukocytosis with a left shift at presentation, and increased CRP level (16), which met the SIRS criteria, had worsened before the start of the steroid therapy. We postulated that SIRS was caused by SS because no other systemic diseases or infections were confirmed.

SIRS is considered an important extracutaneous disorder associated with SS, and it can be fatal in humans without proper treatment (4–6). Some reports also described patients with sudden onset of skin lesions and SIRS worsening without any evidence of infection, who were treated effectively with high-dose steroids (17, 18). However, to our knowledge, there are no reports of cases of SIRS induced by SS in veterinary medicine. In this case, although there is no evidence supporting that a bacterial infection had a causative role in dermatosis, the accompanying fever and peripheral leukocytosis suggested a systemic inflammatory process (19). SS is a rare condition; however, this diagnosis should be considered in cases with typical skin lesions and SIRS without evidence of infection (5).

The SS lesions, if untreated, may persist for weeks or months (1). Although there are no established treatment protocols in veterinary medicine, systemic corticosteroids are the therapeutic mainstay for SS. Other first-line agents in human medicine include potassium iodine and colchicine (1). To date, systemic steroids and immunosuppressive agents, such as cyclosporine or mycophenolate mofetil, have been used for maintenance in veterinary practice (2, 10, 12, 13). Clinical cutaneous or extracutaneous manifestations, in this case, responded effectively to an immunosuppressive dose of prednisolone; however, the skin lesions recurred after tapering of the prednisolone with cyclosporine. As a second-line agent, cyclosporine can be used as a monotherapy or in combination with steroids, and in one veterinary report, remission was achieved after a long-term therapy with cyclosporine (13). However, the therapeutic efficacy cyclosporine alone may not have been sufficient in this case. Mycophenolate mofetil was also used as a steroid-sparing agent, and the clinical signs were controlled for eight months (2). However, there are no validated secondary agents that can replace steroids to date. Therefore, alternatives for long-term management are needed.

Patients with suspected drug-induced SS have a poor prognosis (11, 15), while most cases with the classical SS form tend to achieve complete remission or remission following recurrence (2, 10, 12–14). However, SS reports in veterinary medicine are scarce; thus, it is challenging to estimate the remission or recurrence rates or predict prognosis.

In summary, canine sterile neutrophilic dermatosis or SS is an extremely rare disease, which is challenging to diagnose due to the various extracutaneous manifestations. Furthermore, it is important to recognize SIRS as a severe uncommon extracutaneous SS manifestation to avoid delays in instituting systemic corticosteroid treatment because SIRS can progress to multiple organ dysfunction syndrome and death.

## Data Availability Statement

The original contributions presented in the study are included in the article/supplementary material, further inquiries can be directed to the corresponding author/s.

## Author Contributions

AC, HB, SS, YK, YJ, K-WC, D-IJ, and DY collected clinical data. AC, J-EH, DK, and DY drafted and revised histopathology. AC and DY drafted and revised the manuscript. All authors contributed to the article and approved the submitted version.

## Funding

This work was supported by the Basic Science Research Program through the National Research Foundation of Korea (NRF) and funded by the Ministry of Science, ICT & Future Planning under Grant NRF-2020R1C1C1008675.

## Conflict of Interest

The authors declare that the research was conducted in the absence of any commercial or financial relationships that could be construed as a potential conflict of interest.

## Publisher's Note

All claims expressed in this article are solely those of the authors and do not necessarily represent those of their affiliated organizations, or those of the publisher, the editors and the reviewers. Any product that may be evaluated in this article, or claim that may be made by its manufacturer, is not guaranteed or endorsed by the publisher.

## Abbreviations

SIRS: systemic inflammatory response syndrome
SS: Sweet's syndrome or sterile neutrophilic dermatosis.
